# Mindfulness-based interventions to support wellbeing of adults in low socio-economic settings: a realist review

**DOI:** 10.1186/s12906-023-04263-7

**Published:** 2024-01-24

**Authors:** Sarah Foale, Yvonne Botma, Tanya Heyns

**Affiliations:** 1 Department of Psychiatry, Faculty of Medicine and Health Sciences, University of Stellenbosch, Stellenbosch, South Africa; 2 School of Nursing, University of the Free State, Bloemfontein, South Africa; 3 Department of Nursing Science, Faculty of Health Sciences, University of Pretoria, Pretoria, South Africa

**Keywords:** Adapt, Low income, Low resource, Low socio-economic, Mindfulness, Mindfulness-based intervention, Mindfulness-based stress reduction, Program theory, Realist review, Wellbeing

## Abstract

**Background:**

Mindfulness as a modality involves training the innate human capacity for present-moment awareness with a view to cultivating a more harmonious and integrated life experience, especially in the face of hardship. Over the past four decades, the field of mindfulness has grown rapidly. Despite a substantial body of literature outlining the many benefits of mindfulness practice within a range of contexts and populations, the authors noticed that studies addressing the adaptation, application and value of mindfulness-based interventions (MBIs) for adults within socio-economically challenged setting were scant. To address this gap, we conducted a realist review of studies pertaining to MBIs within low socio-economic settings, to determine the extend and nature of research in this sector and culminating in a program theory which may be useful for the design of interventions going forward.

**Methods:**

We selected realist review as the methodology as it is well suited to investigating the complex nature of social interventions. The value of realist review is that the exploration of the causal relationships between the mechanisms (M) within a specific context (C) towards particular outcomes (O) offers a deeper understanding of the intervention which may assist in more effective delivery going forward. The review follows the guidelines presented by the Realist and Meta-narrative Evidence Synthesis – Evolving Standards project.

**Results:**

Of the 112 documents identified, 12 articles met the inclusion criteria. Of these 12 studies, 10 were conducted in the United States, with little representation across the rest of the globe. The interventions described in these articles were varied. We identified mechanisms that offered beneficial outcomes for participants across a range of contexts, with indications of how interventions might be adapted towards greater accessibility, acceptability, and feasibility within communities.

**Conclusion:**

By reviewing the various programs in their respective contexts, we developed a program theory for implementing socio-culturally adapted MBIs in low socio-economic settings. In the future, this program theory could be tested as a means to create a sense of wellbeing for people living in low socio-economic settings.

**Supplementary Information:**

The online version contains supplementary material available at 10.1186/s12906-023-04263-7.

## Highlights

• Minimal research found in the field of MBIs within low socio-economic settings

• Available studies were analysed and assessed against the “gold standard” of MBSR

• A program theory was developed for adapting MBIs for low socio-economic settings

**Supplementary Information:**

The online version contains supplementary material available at 10.1186/s12906-023-04263-7.

## Introduction

Mindfulness is an innate and universal human capacity that allows for clear thinking and open-heartedness as a means to compassionately alleviate suffering [1]. Mindfulness may be further developed by regularly engaging in formal practices – such as sitting or walking practices – or informal activities – such as eating or washing hands – that ground our attention in both the inner and outer experiences of everyday activities as they occur [2]. These practices center on becoming intentionally aware of one’s thoughts, feelings, and bodily sensations [2–6]. As a parallel process, mindfulness practices focus on cultivating the attitudinal qualities of non-judgement, patience, beginner’s mind, trust, non-striving, acceptance, and letting go [2].

Mindfulness as a modality may be broadly described as an integrated mind–body psycho-spiritual approach originating in the wisdom traditions of Asia, most specifically within Buddhist culture [7]. The term ‘mindfulness’ is however used across a range of disciplines, with each field offering definitions based on the theoretical, methodological and conceptual traditions of the particular discipline [8]. As this review uses the Mindfulness-Based Stress Reduction programme founded by microbiologist Dr Jon Kabat-Zinn as the “gold standard” for contemporary western mindfulness practice, the authors have used Kabat-Zinn’s definition of mindfulness to inform the research: “paying attention in a particular way: on purpose, in the present moment, and non-judgmentally” [2]. It is important to recognize that while MBSR was designed to be appropriate for contemporary, diverse populations, at no time was the intention to divorce the practices from their Buddhist origins. Instead, the capacity to intentionally settle the attention on present-moment awareness within the context of a particular attitudinal approach was offered in a way that recognizes the universality of mindfulness as an expression of our shared humanity [2].

Contemporary mindfulness as an intervention and field of research originated in 1979 when Kabat-Zinn developed the mindfulness-based stress reduction (MBSR) program at the University of Massachusetts Medical School [2]. This program is now used internationally as a manualized, experiential 8-week intervention that involves training in a specific range of formal and informal mindfulness practices [9]. The program enables participants to cultivate mindful awareness in everyday life as a means to develop coping strategies for dealing with stressful and difficult conditions and alleviating both psychological and physiological suffering, leading to an enhanced sense of wellbeing [2–5, 7, 10–12].

Following the success of MBSR, mindfulness has become increasingly popular worldwide amongst clinicians, educators and the general public as a means to cultivate a sense of integrated well-being [2, 3, 5, 10, 13–19]. Despite the substantial body of literature outlining the many benefits of mindfulness practice within a range of contexts and populations, studies addressing the adaptation, application and value of mindfulness-based interventions (MBIs) for adults in low socio-economic settings are scant. Until now, most mindfulness research has been conducted amongst what may be referred to as WEIRD populations: western, educated, industrialized, rich and democratic, and particularly white, female, middle- to upper-class individuals [10, 12, 20–22]. This review seeks to address the gap in the literature by establishing the extent and nature of research on mindfulness-based programmes addressing wellbeing with low socio-economic settings, giving rise to the research question: what are the reported mechanisms used in and resultant outcomes of MBIs in low socio-economic settings? These findings culminate in the development of a program theory which may be useful for the design of mindfulness-based programmes going forward, for the benefit of individuals within low socio-economic settings.

In this review, the term ‘low socio-economic’ is used to describe communities or settings that are characterized by some or all of the following factors: poverty or significant low income; harsh physical living conditions including inadequate housing and homelessness; poor access to resources, including education, health-care and employment opportunities; historical disadvantage caused by cultural and racial factors; and displacement for political or economic reasons [23].

## Methods

We conducted a realist review to investigate the complex nature of social interventions, in this case, MBIs for wellbeing in low socio-economic settings. The realist review provided an opportunity to unpack the theories embedded in descriptions of interventions and explore the impact of context on the effectiveness of interventions [24].

Due to the complexity of social interventions, traditional forms of review are unlikely to offer complete or meaningful results, as they do not consider the effects of differing contexts on outcomes [24, 25]. Rather than simply seeking to establish the efficacy of an intervention, realist review offers an explanation of how the intervention worked [26]. Realist reviews explain the causal relationships between mechanisms (M) within a specific context (C) which will have particular outcomes (O): M + C = O [24, 25]. Realist reviews offer a deeper understanding of the intervention which may assist in more effective delivery going forward [24].

We used the methodology for realist review outlined by Pawson et al. (2005) and reported our results following guidelines by Wong et al. as part of the Realist and Meta-narrative Evidence Synthesis – Evolving Standards (RAMESES) project [27]. The first four stages of a realist synthesis are: 1) clarifying the search strategy, including identifying the review question and the nature of the intervention, as well as refining the purpose of the review and exploring program theory; 2) searching for evidence and selecting studies for inclusion; 3) appraising primary studies and extracting data; and 4) synthesizing the data to refine program theory and determining what works for whom, how, and under what circumstances [24].

### Defining and clarifying the scope of research

#### Identifying the review question

Using the M + C = O formula, we defined the review question as follows: within low socio-economic settings (C) what are the mechanisms (M) and outcomes (O) of MBIs for wellbeing? This question was refined into three further questions: 1) within low socio-economic settings, in what contexts do MBIs designed to enhanced wellbeing take place; 2) what are the key mechanisms that benefit or hinder these MBIs; and 3) what are the outcomes of these MBIs?

#### Clarify the purpose of the review

We conducted this review to establish the feasibility, acceptability, and accessibility of MBIs conducted in low socio-economic settings. This forms part of a larger vision to provide both economically and methodologically sound offerings in low income, low resource communities, toward facilitating social transformation and upliftment.

#### Developing the program theory

To build the initial program theory, we conducted a preliminary search of the literature to determine the availability of literature [27]. We searched Google Scholar using combinations of the search terms “mindfulness”, “mindfulness-based intervention”, “MBI, “mindfulness-based stress reduction”, “MBSR”, “low income”, “low resource”, “poverty”, “underserved” and “socio-economically challenged”. This broad reading provided the foundation for developing the preliminary program theory; that MBIs for wellbeing, when delivered using certain mechanisms, may benefit adult participants in low socio-economic settings.

### Search strategy

#### Inclusion and Exclusion Criteria

To determine inclusion and exclusion criteria, we followed the population-concept-context (PCC) methodology [28]. We included literature on the adult population, including older adults, and excluded articles focusing on adolescents and children under 18. As per the aim of this review, we retrieved articles focusing specifically on the cultivation of wellbeing. We excluded articles that addressed psychological or medical pathologies, as well as therapeutic interventions specifically addressing drug and alcohol addiction, as mindfulness literature (although limited) has cautioned against including individuals in MBPs if they have a diagnosis of schizophrenia, bipolar disorder, obsessive–compulsive disorder, post-traumatic stress disorder [29], suicidality, untreatable psychosis, clinical depression, social anxiety and other major psychiatric diagnoses [30, 31]. We included articles pertaining to mindfulness interventions that treated tobacco smoking or weight loss/gain (where there were no clinical diagnoses of anorexia, bulimia or obesity), domestic violence, and trauma as these are generic in many low socio-economic populations. and are directly associated with wellbeing. In terms of concept, we only included articles that mentioned mindfulness-based interventions or Mindfulness-based Stress Reduction in the title or abstract. Regarding context, we only included articles that specifically discussed MBIs in low income or low resource communities or settings, as well as populations defined as under-served, poor, marginalized, or disadvantaged. We excluded research conducted in any other settings, or articles that did not specify the setting. We included only peer-reviewed manuscripts, written in English. We excluded non-primary research, books, reviews, summaries, and protocols.

#### Literature search

An experienced librarian conducted the literature search across a range of databases using the keywords provided by the authors. Whereas the preliminary search represented a broad reading of the field, this formal search is a reproducible and unbiased collection of sources which may be used to test the program theory [25].

#### Search string

The librarian used the following search string: "mindfulness-based intervention*" or MBI or "mindfulness-based stress reduction*" or MBSR or "mindfulness-based program*" (specifically excluding “Maslach” as MBI can also be “Maslach Burnout Inventory”); AND “low income" or low-income" or "low resource*" or low-resource* or poor or poverty* or "limited resource*" or underprivileg* or Disadvantag* or Underserv* or "socio-economical* challeng*" or marginali* or "low econom*" or low-econom*. The librarian mainly searched the EBSCOHost database, as follows: Academic Search Ultimate, Africa-Wide Information, APA PsycArticles, APA PsycInfo, CAB Abstracts, CINAHL with Full Text, ERIC, GreenFILE, Health Source—Consumer Edition, Health Source: Nursing/Academic Edition, Humanities Source Ultimate, MEDLINE, Sociology Source Ultimate, SPORTDiscus with Full Text, OpenDissertations.

**Fig. 1:**
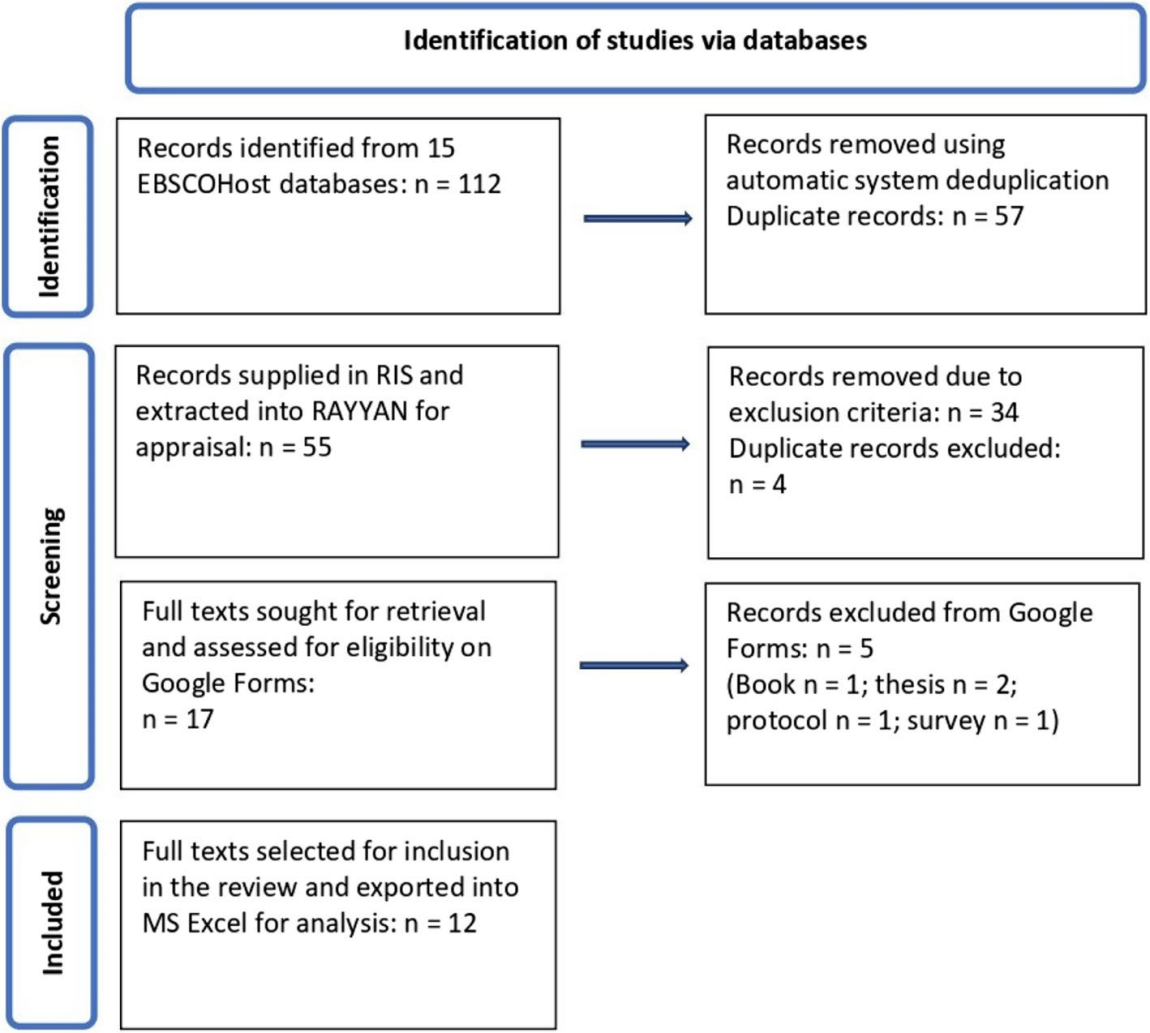
PRISMA flow diagram showing the process followed to retrieve articles focusing on mindfulness-based interventions (MBIs) to promote wellbeing of adults in low socio-economic settings

Following identification (*N* = 112) and deduplication (*n* = 57) of the records (titles and abstracts), the librarian supplied the search results (*n* = 55 records) in research information systems (.ris) format. We then imported the records into RAYYAN (www.rayyan.ai) for full-text screening. After identifying and removing further duplicates (*n* = 4), the authors independently screened the records using the PCC inclusion and exclusion criteria. The authors then met to discuss any disputed articles and subsequently excluded 34 articles. Of the remaining 17 articles, we excluded five articles (two due to duplication, including a journal article based on a thesis, one that was not relevant, one due to inappropriate research design and one because it was a survey), leaving 12 articles to be included in the analysis (Fig. 1).

#### Data appraisal and analysis

Using both the review question and the preliminary program theory as a guide, we designed a data extraction tool using Google Forms. The data extraction tool had the following categories: author; title; year published; journal; aim; purpose of study; research design; population; sampling method; sample size; context; mechanisms/intervention; tools; training and experience of facilitator; outcomes; lessons; and recommendations for further research. Data were then extracted from the articles using the data extraction tool. The authors collectively confirmed the data by checking the charted data against each article. Any discrepancies were discussed and resolved.

## Results

### Study characteristics

Of the 12 articles, 10 were published in the past decade. There were no articles prior to 2007 that specifically addressed MBSR programs or MBIs in low socio-economic settings. Most (*n* = 10) of the studies were conducted in the United States (USA) [32–41]. Only two studies were conducted elsewhere: one in Canada [42] and one in Belgium [16]. The large number of studies conducted in the USA is consistent with current findings that since 1996, 90% of all research on mindfulness has been conducted in USA and the United Kingdon (UK), with no specific research within low socio-economic settings occurring in other regions [43].

### Methodologies

Of the 12 articles, two were randomized controlled trials [35, 41], two had a pre-posttest design [16, 38], four were qualitative [33, 39, 40, 42] and five did not report study design.

### Population

Seven studies focused solely on women [32, 33, 35–37, 40, 41]. Two studies only included pregnant women [36, 41]. Four studies did not mention gender. One article included older adults, between 60 and 90-years-old [40]. Two articles targeted populations that had experienced trauma [35, 37].

### Sampling process

Five articles did not describe sampling or inclusion/exclusion criteria for selecting participants [33, 34, 38, 40, 41]. In the remaining eight studies, four mentioned inclusion and exclusion criteria but did not report sampling [32, 36, 39, 44], one reported using volunteers [16]; one used convenience sampling [42] and one used randomized sampling [35]. Only two articles mentioned a recruitment strategy [42, 44].

### Sample size

The sample sizes varied considerably. Two studies had a sample size of over 100: 106 and 215 participants respectively [35, 36]. The other 10 studies had sample sizes ranging from 10 to 56 participants. In terms of attrition, three studies reported attrition between 30 and 60% [35, 36, 44], while four reported attrition between 60 and 84% [16, 32, 41, 42]. The remaining six studies did not mention attrition.

### Research tools

Various research tools were used in these studies. The only tools that were used in more than one study were the Self-Compassion Scale (*n* = 2), the Pittsburgh Sleep Quality Index (*n* = 2), the Perceived Stress Scale (*n* = 3), the Five Facet Mindfulness Questionnaire (*n* = 2) and focus groups (*n* = 3). Five studies used demographic questionnaires [36, 38, 39, 44]. The studies that were concerned with acceptability and feasibility all used different tests [35, 38, 42, 44]. Two studies measured physiological biomarkers: one for cortisol [41] and one reported immunological data [44].

### Context

The articles reported varied contexts in which the interventions were offered. One study did not mention the actual context, other than the town where the study was conducted [42]. Two programs were run in shelters for women, one particularly for those experiencing domestic violence and homelessness [33, 35]. Six interventions were run either in clinics or hospitals, including a women’s clinic and a university hospital [32, 34, 36, 39, 41, 44]. One intervention was conducted amongst residents at a low-income senior housing facility [40]; one was run amongst paraprofessionals working within a pre- and primary school setting for children demonstrating emotional, behavioral and academic risk [38]; and one was offered to visitors to public social and health facilities [16]. Only four studies reported the actual geographic location of the intervention [16, 34, 40, 42].

### Facilitator training and experience

Five articles did not mention the training and experience of facilitators. One article mentioned that the facilitator had a well-established personal practice and was experienced in adapting mindfulness practices for high-risk youth yet did not refer to any training in mindfulness facilitation [38]. Another article described the facilitators demographically and academically but did not mention experience or qualifications in mindfulness [34]. Two studies described facilitators who were experienced and had received mindfulness facilitator training – one facilitator had online training in the program [41] and, the other facilitator had weekly supervision in the program [36]. Three studies specifically mentioned the qualifications of the facilitators, including that all facilitators were experienced [16, 35, 44].

### Mechanisms

Due to the decades-long history of MBSR and the substantial body of research related to its application in a variety of contexts and settings, MBSR may be viewed as the “gold standard” against which other MBIs might be assessed [4]. Alongside this foundational structure, we need to consider how MBSR might be adapted to different situations to meet ethical obligations of providing culturally sensitive service delivery [12]. Table 1 indicates how the 12 articles included in this review retained the core principles of the MBSR “gold standard” (the “warp”) while adapting the interventions to be more context-sensitive (the “weft”) [4]. One study was a straight MBSR [44], seven were adapted MBSRs [16, 32–35, 40, 42]; three were MBIs that differed from MBSR on certain key elements [36, 38, 41]; and one article tested the effects of two 2-h focus groups on wellbeing [39].

**Table 1:** Comparison of reviewed articles on mindfulness-based interventions (MBI) with the practice of mindfulness-based stress reduction (MBSR) [4]

Author	Description	Number of weeks	Session length	Retreat	Additional themes	Adapted structure	Additional comments	Facilitator training
Bermudez et al. (2013) [33]	Adapted MBSR	10	Not specified	Yes, after session 8	Loving-kindness; music; mindful planning	Slight variation in session progression	Not specified	Not specified
Abercrombie et al. (2007) [32]	Adapted MBSR	6	2 h	Not specified	Related to the context of the program, i.e., pap smears	Reduced number of sessions and shorter sessions	Not specified	Not specified
Bhambhani and Gallo (2022) [34]	Adapted MBSR	16	1 h	Not specified	Trauma-informed cultural humility approach	16 weekly sessions divided into four modules Shortened sessions and practices Language adapted	More directive than MBSR in terms of inquiry, homework review and psychoeducation	Facilitated by the researchers Qualifications not mentioned
Zhang and Emory (2015) [41]	MBI – Mindful Motherhood	8	Not specified	Not specified	Not specified	Not specified	Factors that contribute to treatment barriers need to be considered Need to improve strategies encouraging participation and adherence, including cultural relevance	Facilitated by an advanced PhD student in clinical psychology Online training in the intervention Experience and qualifications not given
Van der Gucht et al. (2015) [16]	Adapted MBSR	8	90 min	Not specified	Psychoeducation	Shortened sessions	Possibly increase participation rates by using financial incentives	Trained mindfulness facilitators (*n* = 2): clinical psychologist (*n* = 1) and social worker (*n* = 1) Qualification not discussed Both experienced
Szanton et al. (2011) [40]	Focus group study of adapted MBSR	8	Shortened sessions	Not specified	Not specified	More sitting practice—less mindful movement and walking Shortened sessions	Not specified	Not specified
Spears et al. (2017) [39]	120-min focus group after two 5min practices	Not applicable	2 h	Not applicable	Reflection on mindfulness and specific practices, including appeal, relevance, language, acceptability, and perceived feasibility	Short practices (*n* = 2) on mindful breathing and body scan	Suggestions offered regarding how practice might be integrated into daily life; preparing participants for the possibility of experiencing discomfort; creating an atmosphere of safety and support	Not specified
Hick and Furlotte (2010) [42]	Adapted MBSR – integrated with Radical Mindfulness Training (RMT)	Not specified	Not specified	Not specified	Focus on sociological education to understand the roots of poverty	Not specified	Radical mindfulness training with core elements of MBSR. engages participants at the personal, interpersonal, and structural levels	Not specified
Jacobs et al. (2017) [38]	MBI	6 sessions over 8 weeks	90 min	Not specified	Muscle relaxation / no mindful eating	Restructured MBSR; 6 weeks instead of 8; shorter sessions; and shorter practices averaging 5 min in duration	Not specified	Facilitator not specifically trained in mindfulness but had 6 years personal practice as well as experience in adapting mindfulness practices for high-risk youth in urban communities
Dutton et al. (2013) [35]	Adapted MBSR	10 sessions	90 min	5 h—between session 8 and 9	Additional mindfulness practices including loving-kindness and mindfulness listening exercises	Pre-program orientation sessions with the MBSR instructor; changing the beginning of each session; changing the sequence of sessions; shorter and fewer sessions; logistical support for participants; and ensuring voluntary participation	Minimally adapted MBSR—more accessible to the population	A trained and experienced female psychiatric nurse (MSN) with teacher-level training in MBSR
Gallegos et al. (2015) [37, 44]	MBSR	8	2 h plus	4 h	Nil reported	Nil reported	Program was run according to the MBSR manual developed by the University of Mass. Center for Mindfulness	Program delivered by experienced teacher with advanced training
Felder et al. (2018) [36]	MBI	8	2 h	Not specified	Mindful eating specifically	Similar to MBSR i.e., 8 × 2-h sessions	Aim was to reduce overeating as a respoe to stress	Facilitated by two experienced mindfulness teachers who received weekly supervision

### Outcomes

Of the 12 articles reviewed, one article reported that participants believed that mindfulness practice might improve their mental and physical health but did not report clear outcomes [39]. The outcomes of the remaining 12 articles are reported in Table 2. We analyzed the outcomes deductively by aligning the outcomes with mindfulness theory. Mindfulness practice involves understanding our lived experiences by recognizing the nature of our thoughts (cognitive processes), emotions and physical sensations (physiological elements), particularly when experiencing stress. In addition to the outcomes described in the Table 3, one article reported that modifying the MBI in particular ways improved outcomes for people facing extreme poverty and associated difficulties [42]. Zhang and Emory [41] reported that attending more mindfulness sessions reduced depressive symptoms one-month post-intervention. Two articles reported on the acceptability of their initiatives [38, 40], one article reported on feasibility [42], and two articles reported on both acceptability and feasibility [35, 44].

**Table 2:** Outcomes reported in the reviewed articles describing mindfulness-based interventions (MBI) for wellbeing in low socioeconomic settings

Category	Outcomes	Abercrombie et al. (2007) [32]	Bermudez et al. (2013) [33]	Bhambhani and Gallo (2022) [34]	Felder et al. (2018) [36]	Gallegos et al. (2015) [37, 44]	Hick and Furlotte (2010) [42]	Jacobs et al. (2017) [38]	Szanton et al. (2011) [40]	Van der Gucht et al. (2015) [16]	Zhang and Emory (2015) [41]	Total
**Physiological**	Improved health / pain management	Yes		Yes								2
	Improved sleep quality							Yes				1
**Cognitive**	Improved focus			Yes								1
	Increased awareness / self-awareness		Yes	Yes								2
	Increased self-kindness / self-compassion		Yes				Yes					2
	Improved self-advocacy/ assertive communication / capacity to affect changes to challenges		Yes	Yes			Yes					3
	Increased capacity to overcome personal trauma		Yes									1
	Improved anger management		Yes	Yes					Yes			3
	Improved coping skills relating to depression and medical procedures								Yes			1
	Improved eating habits			Yes								1
	Improved overall subjective wellbeing						Yes			Yes		2
	Decreased reactivity									Yes		1
	Decreased over-generalization									Yes		1
	Decreased self-judgement / judgement			Yes			Yes					2
**Emotional**	Improved emotional regulation		Yes			Yes						2
	Improved emotional health			Yes								1
	Increased serenity			Yes								1
	Decreased emotional dysregulation					Yes						1
	Decreased emotional exhaustion							Yes				1
	Decreased depression			Yes		Yes				Yes	Yes	4
	Decreased anxiety					Yes				Yes		2
**Interpersonal**	Increased capacity for socialization/ connection		Yes				Yes					2
	Improved quality of relationships/ interpersonal functioning		Yes	Yes								2
	Decreased isolation						Yes					1
**Stress**	Decreased stress	Yes				Yes				Yes		3
	Decreased perceived stress				Yes			Yes				2
	Decreased post-traumatic stress symptoms					Yes						1
**Mindfulness**	Increased mindfulness (capacity for awareness and description of experiences as well as non-judgement and non-reactivity)					Yes				Yes		2

**Table 3:** Components of the program theory for a mindfulness-based intervention (MBI) for adults in a low socio-economic setting

WHAT (Adapted MBI)		HOW	OUTCOME
Mechanisms	Length of program	Modified according to requirements of community	More acceptable, accessible and feasible = less attrition and more engagement = improved general outcomes, in particular wellbeing as determined by biological and psychological parameters, and measured using a consistent battery of tests
	Length of sessions	Shortened	
	Length of practices	Shortened	
	Trauma sensitive	Practices and other elements adjusted according to participants	
	Cultural resonance	In terms of metaphors and practices used	
	Language	Easily understood by participants	
	Literacy	Accommodations made for those not literate	
Facilitator	Training	Relevant training to design and deliver MBIs	
	Embodiment	Presence models the attitudes of mindfulness	
	Experience	Familiar with dynamics of group work specific to the participants	
	Trauma-aware	Trained to determine indications of trauma and mediate appropriately	
	Demographic	Culturally similar to participants	

### Recommendations to improve MBI outcomes in terms of acceptability, accessibility and feasibility

The articles reviewed recommended several improvements for MBIs in the future. The specific barriers to participation in MBIs were noted as time conflicts, financial constraints [32, 39], lack of childcare, transport, work scheduling, health problems, death of a family member [16], unsafe neighborhoods and unsafe housing [39].

Suggestions for reducing attrition varied depending on the population’s cultural practices, values and needs – particularly for women whose lives tend to be more unpredictable and demanding [32]. Women might be encouraged to participate if there is support in terms of meals, childcare, transport and weekly reminders, as well as financial incentives and offering classes at multiple times during the week [16, 32, 35, 41, 44].

Interventions could be adapted to increase accessibility by delivering the program in the population’s mother-tongue or offering translations and using examples from participants’ lives [32]. Community members could be involved in various ways, such as training community members to be facilitators [35]. This would ensure that facilitators are from a similar cultural background to the participants, especially if they share similar trauma [33, 34, 41]. Facilitators should be appropriately trained in MBI delivery [35]. Participation could also be improved if there were fewer and shorter sessions without compromising positive outcomes [38, 42]. MBIs could be integrated into home practice in such a way that it does not increase daily pressure and stress [35].

One-on-one pre-interventions sessions could help to clarify participants’ understanding and expectations of the program [35]. Participants and facilitators should continuously engage to address concerns and facilitate involvement [35, 39].

Ten articles recommended further research, including replicating existing studies with optimal retention [41]; examining the biological and bio-behavioral parameters of health, stress reactivity, and resilience [16, 44] investigating the dose of practice on response [38]; and exploring the relationship between mindfulness and physical health in low-income populations, including amongst older adults [40].

Several studies suggested larger samples and the use of control groups to confirm the findings of pilot studies, as well as randomized control and longitudinal studies [35, 42]. Studies should ensure bias-free assessment by including larger assessment batteries [38].

## Discussion

The results of this review indicate that no studies have been published on the role of MBIs to cultivate wellbeing in low socio-economic settings beyond the “first world” and the USA in particular. This leaves an open field for research on the value of MBIs in low socio-economic settings the world over. The reviewed articles were varied in terms of methodologies, populations, sampling processes, sample sizes, context, training and experience of facilitators, and the mechanisms used. We, therefore, could not compare studies or draw any overall conclusions from the data. What did emerge from the data, however, was that adapting the mechanisms of programs was fundamental for increasing feasibility, accessibility, and acceptability, for example by modifying or reducing the length of the program, sessions and practices; providing manageable home-practice components; and most importantly ensuring that facilitators are appropriately trained and qualified. Most articles recommended strategies to adapt these mechanisms.

To be identified as MBIs, programs need to have certain key features, including upholding the core principles and underlying tenets of mindfulness. Practices should involve sustained intensive training grounded in present moment focus, decentering and an approach orientation [4]. MBSR can be seen as the “gold standard” of MBIs and can be adapted to accommodate the particular needs of participants within their unique contexts. Most of the programs reviewed in this article were described as either MBSRs or adaptations thereof. Few studies defined the actual content of the sessions and the extent to which they incorporated the features of mindfulness. Future research would need to clarify why the intervention should be considered an MBI.

Considering how fundamental the training and experience of the facilitator is to the integrity of the program, it is notable that five articles did not mention the facilitators’ qualifications. Only four articles indicated that the program facilitators were appropriately trained. The remaining articles suggested that while facilitators were knowledgeable and experienced, they were not specifically trained in mindfulness. A key requirement of effective MBIs is that they are developed and run by qualified and experienced facilitators, who are not only grounded in the principles and practices of mindfulness, but also well-versed in adapting material and practices to the requirements of specific populations.

The initial program theory that emerged from the preliminary literature review suggested that MBIs directed towards enhancing wellbeing, when delivered using certain mechanisms, may benefit adult participants in low socio-economic settings. Based on the components of program theory shown in Table 3, we hypothesize that the sense of wellbeing of individuals in low socio-economic settings will improve following participation in an authentic MBI when it has been appropriately adapted through a process of community negotiation and implemented by a trained and competent facilitator. In conclusion we propose that the hypothesis is tested as part of future research within LSES across a broader geographic base.

## Limitations

Our findings may be limited by our search strategy. We searched for specific keywords mentioned in the articles’ titles and abstracts. Any article that did not specifically list those keywords would have been automatically excluded, especially articles published in languages other than English. In addition, the sample size was small and largely US-based because this was all that the search revealed. While this certainly limits the generalisability of the findings, it serves as a call for more research into the implementation and outcomes of mindfulness-based well-being initiatives in low socio-economic settings worldwide. A further limitation is that the quality of studies was generally poor due to inadequate methodological information.

## Conclusion

The articles reviewed here offer recommendations for how MBIs might be adapted to best serve adults in low socio-economic settings—albeit within “first world” environments. By reviewing the mechanisms and outcomes of the various programs in their respective contexts, we developed a program theory for implementing socio-culturally adapted MBIs in low socio-economic settings, including “third world” or developing countries characterized by varied economies, languages, and cultural practices.

This review may be seen as rigorous in that 1) the literature search was done by a qualified and experienced librarian; 2) multiple databases were searched; 3) all three authors participated in the selection, review, checking and synthesis of the articles, which was 4) conducted iteratively while 5) adhering to the RAMESES reporting standards [27].

Future research could test this program theory in a variety of settings, particularly low socio-economic settings in countries other than the USA. The program theory will ensure the conceptual integrity of MBIs as a modality and also provide a sound platform for adapting and comparing MBIs. The program theory presented here suggests a means to deepen best practice and offers a potentially effective approach to reducing suffering and increasing a sense of wellbeing for adults in low socio-economic settings.

### Supplementary Information

**Additional file 1.** 

## Data Availability

All data generated or analysed during this study are included in this published article and its supplementary information files.

## Abbreviations

C: Context
MBI: Mindfulness-based intervention
O: Outcome
UK: United Kingdom
M: Mechanism
MBSR: Mindfulness-based stress reduction
RAMESES: Realist and Meta-narrative Evidence Synthesis-Evolving Standards
USA: United States of America

## References

[1] Dobkin, P.L. Mindfulness and Whole Person Care. In: Hutchinson, T. (eds) Whole Person Care. Springer: New York. 2011. 10.1007/978-1-4419-9440-0_7.

[2] Kabat-Zinn J. Full catastrophy living: Using the wisdom of your body and mind to face stress, pain, and illness. New York: Bantam Books; 2013.

[3] Zhang D, Lee EKP, Mak ECW, Ho C, Wong SYS (2021). Mindfulness-based interventions: an overall review. Br Med Bull.

[4] Crane RS, Brewer J, Feldman C, Kabat-Zinn J, Santorelli S, Williams JMG, Kuyken W (2017). What defines mindfulness-based programs? The warp and the weft. Psychol Med.

[5] Teasdale J, Segal ZV. The mindful way through depression: freeing yourself from chronic unhappiness. New York: Guilford Press; 2007.

[6] Baer RA (2003). Mindfulness training as a clinical intervention: a conceptual and empirical review. Clin Psychol Sci Pract.

[7] Van Gordon W, Shonin E (2020). Second-generation mindfulness-based interventions: Toward more authentic mindfulness practice and teaching. Mindfulness.

[8] Nilsson H, Kazemi A (2016). Reconciling and thematizing definitions of mindfulness: The big five of mindfulness. Rev Gen Psychol.

[9] Santorelli, S. F., Kabat-Zinn, J., Blacker, M., Meleo-Meyer, F., & Koerbel, L. Mindfulness-based stress reduction (MBSR) authorized curriculum guide. Center for Mindfulness in Medicine, Health Care, and Society (CFM). University of Massachusetts Medical School. 2017. Available from: https://www.tarkustekool.ee/wp-content/uploads/2021/09/CFM-Teaching-UMass-MBSR-Curriculum-Teaching-Guide-2017.pdf Accessed on 16 March 2023.

[10] Aizik-Reebs A, Yuval K, Hadash Y, Gebreyohans Gebremariam S, Bernstein A (2021). Mindfulness-based trauma recovery for refugees (MBTR-R): Randomized waitlist-control evidence of efficacy and safety. Clin Psychol Sci.

[11] Cotter EW, Jones N (2020). A review of Latino/Latinx participants in mindfulness-based intervention research. Mindfulness.

[12] Wasson RS, Luberto CM, Murthi M, McDonald SB, Pallerla H, Novak BK, Cotton S (2020). Feasibility and acceptability of a community-based modified mindfulness-based stress reduction program for the under- and unemployed. Glob Adv Health Med.

[13] Pillay K, Eagle G (2021). The case for mindfulness interventions for traumatic stress in high violence, low resource settings. Curr Psychol.

[14] Querstret D, Morison L, Dickinson S, Cropley M, John M (2020). Mindfulness-based stress reduction and mindfulness-based cognitive therapy for psychological health and well-being in nonclinical samples: a systematic review and meta-analysis. Int J Stress Manag.

[15] Goleman D, Davidson R (2017). The science of meditation: How to change your brain, mind and body.

[16] Van der Gucht K, Takano K, Van Broeck N, Raes F (2015). A mindfulness-based intervention for economically disadvantaged people: effects on symptoms of stress, anxiety, and depression and on cognitive reactivity and overgeneralization. Mindfulness.

[17] Khoury B, Lecomte T, Fortin G, Masse M, Therien P, Bouchard V, Chapleau MA, Paquin K, Hofmann SG (2013). Mindfulness-based therapy: a comprehensive meta-analysis. Clin Psychol Rev.

[18] Siegel DJ (2007). Mindfulness training and neural integration: differentiation of distinct streams of awareness and the cultivation of well-being. Social Cognitive and Affective Neuroscience.

[19] Siegel RD. The mindfulness solution: everyday practices for everyday problems. New York: Guilford Press; 2009.

[20] Castellanos R, Yildiz Spinel M, Phan V, Orengo-Aguayo R, Humphreys KL, Flory K (2020). A systematic review and meta-analysis of cultural adaptations of mindfulness-based interventions for Hispanic populations. Mindfulness.

[21] Roth B, Robbins D (2004). Mindfulness-based stress reduction and health-related quality of life: Findings from a bilingual inner-city patient population. Psychosom Med.

[22] Woidneck M, Pratt K, Gundy J, Nelson C, Twohig M (2012). Exploring cultural competence in acceptance and commitment therapy outcomes. Prof Psychol Res Pract.

[23] Fritz F, Tilahun B, Dugas M (2015). Success criteria for electronic medical record implementations in low-resource settings: a systematic review. J Am Med Inform Assoc.

[24] Pawson R, Greenhalgh T, Harvey G, Walshe K (2005). Realist review - a new method of systematic review designed for complex policy interventions. J Health Serv Res Policy.

[25] Clark J, Sweet L, Nyoni S, Ward PR (2020). Improving male involvement in antenatal care in low and middle-income countries to prevent mother to child transmission of HIV: a realist review. PLoS One.

[26] Sinclair S, Kondejewski J, JaggiRoze des Ordons PAL, Kassam A, Hayden KA, Hack TF (2021). What works for whom in compassion training programs offered to practicing healthcare providers: a realist review. BMC Med Educ.

[27] Wong G, Greenhalgh T, Westhorp G, Buckingham J, Pawson R (2013). RAMESES publication standards: realist syntheses. BMC Med.

[28] Peters MD, Marnie C, Tricco AC, Pollock D, Munn Z, Alexander L, McInerney P, Godfrey CM, Khalil H (2020). Updated methodological guidance for the conduct of scoping reviews. JBI Evid Synthesis.

[29] Hanley AW, Abell N, Osborn DS, Roehrig AD, Canto AI (2016). Mind the gaps: are conclusions about mindfulness entirely conclusive?. J Couns Dev.

[30] Binda DD, Greco CM, Morone NE (2022). What Are Adverse Events in Mindfulness Meditation?. Glob Adv Health Med..

[31] Santorelli, S. (Ed.). Mindfulness- stress reduction (MBSR): Standards of practice. Worcester, MA: Center for Mindfulness in Medicine, Health Care & Society, University of Massachusetts Medical School. 2014.

[32] Abercrombie PD, Zamora A, Korn AP. Lessons learned: Providing a mindfulness-based stress reduction program for low-income multiethnic women with abnormal pap smears. Holist Nurs Pract. 2007;21(1):26–34. 10.1097/00004650-200701000-00006 17167329

[33] Bermudez D, Benjamin MT, Porter SE, Saunders PA, Myers NAL, Dutton MA (2013). A qualitative analysis of beginning mindfulness experiences for women with post-traumatic stress disorder and a history of intimate partner violence. Complement Ther Clin Pract.

[34] Bhambhani Y, Gallo L (2022). Developing and adapting a mindfulness-based group intervention for racially and economically marginalized patients in the Bronx. Cogn Behav Pract.

[35] Dutton MA, Bermudez D, Matás A, Majid H, Myers NL (2013). Mindfulness-based stress reduction for low-income, predominantly African American women with PTSD and a history of intimate partner violence. Cogn Behav Pract.

[36] Felder JN, Laraia B, Coleman-Phox K, Bush N, Suresh M, Thomas M, Adler N, Epel E, Prather AA (2018). Poor sleep quality, psychological distress, and the buffering effect of mindfulness training during pregnancy. Behav Sleep Med.

[37] Gallegos A, Lytle M, Moynihan J, Talbot N (2015). Mindfulness-based stress reduction to enhance psychological functioning and improve inflammatory biomarkers in trauma-exposed women: a pilot study. Psychol Trauma.

[38] Jacobs RH, Guo S, Kaundinya P, Lakind D, Klein J, Rusch D, Walden A, Mehta T, Atkins M (2017). A pilot study of mindfulness skills to reduce stress among a diverse paraprofessional workforce. J Child Fam Stud.

[39] Spears CA, Houchins SC, Bamatter WP, Barrueco S, Hoover DS, Perskaudas R (2017). Perceptions of mindfulness in a low-income, primarily African American treatment-seeking sample. Mindfulness.

[40] Szanton SL, Wenzel J, Connolly AB, Piferi RL (2011). Examining mindfulness-based stress reduction: Perceptions from minority older adults residing in a low-income housing facility. BMC Complement Altern Med.

[41] Zhang H, Emory EK (2015). A mindfulness-based intervention for pregnant African-American women. Mindfulness.

[42] Hick SF, Furlotte C (2010). An exploratory study of radical mindfulness training with severely economically disadvantaged people: Findings of a Canadian study. Aust Soc Work.

[43] Baminiwatta A, Solangaarachchi I (2021). Trends and developments in mindfulness research over 55 years: a bibliometric analysis of publications indexed in Web of Science. Mindfulness.

[44] Gallegos AM, Lytle MC, Moynihan JA, Talbot NL (2015). Mindfulness-based stress reduction to enhance psychological functioning and improve inflammatory biomarkers in trauma-exposed women: a pilot study. Psychol Trauma Theory Res Pract Policy.

